# Effect of donor variation on osteogenesis and vasculogenesis in hydrogel cocultures

**DOI:** 10.1002/term.2807

**Published:** 2019-02-08

**Authors:** Iris Pennings, Lukas A. van Dijk, Juliet van Huuksloot, Joost O. Fledderus, Koen Schepers, A. Koen Braat, Edward C. Hsiao, Emilie Barruet, Blanca M. Morales, Marianne C. Verhaar, Antoine J.W.P. Rosenberg, Debby Gawlitta

**Affiliations:** ^1^ Department of Oral and Maxillofacial Surgery and Special Dental Care University Medical Center Utrecht, Utrecht University Utrecht the Netherlands; ^2^ Department of Nephrology and Hypertension University Medical Center Utrecht, Utrecht University Utrecht the Netherlands; ^3^ Department of Cell Biology University Medical Center Utrecht, Utrecht University Utrecht the Netherlands; ^4^ Department of Medicine and the Institute for Human Genetics and the Program for Craniofacial Biology University of California San Francisco San Francisco CA

**Keywords:** coculture model, donor variation, endothelial colony forming cell (ECFC), human induced pluripotent stem cells (iPSC), Matrigel, multipotent mesenchymal stromal cell (MSC), osteogenic differentiation, vasculogenesis

## Abstract

To introduce a functional vascular network into tissue‐engineered bone equivalents, human endothelial colony forming cells (ECFCs) and multipotent mesenchymal stromal cells (MSCs) can be cocultured. Here, we studied the impact of donor variation of human bone marrow‐derived MSCs and cord blood‐derived ECFCs on vasculogenesis and osteogenesis using a 3D in vitro coculture model. Further, to make the step towards cocultures consisting of cells derived from a single donor, we tested how induced pluripotent stem cell (iPSC)‐derived human endothelial cells (iECs) performed in coculture models. Cocultures with varying combinations of human donors of MSCs, ECFCs, or iECs were prepared in Matrigel. The constructs were cultured in an osteogenic differentiation medium. Following a 10‐day culture period, the length of the prevascular structures and osteogenic differentiation were evaluated for up to 21 days of culture. The particular combination of MSC and ECFC donors influenced the vasculogenic properties significantly and induced variation in osteogenic potential. In addition, the use of iECs in the cocultures resulted in prevascular structure formation in osteogenically differentiated constructs. Together, these results showed that close attention to the source of primary cells, such as ECFCs and MSCs, is critical to address variability in vasculogenic and osteogenic potential. The 3D coculture model appeared to successfully generate prevascularized constructs and were sufficient in exceeding the ~200 μm diffusion limit. In addition, iPSC‐derived cell lineages may decrease variability by providing a larger and potentially more uniform source of cells for future preclinical and clinical applications.

## 1 INTRODUCTION

Autologous bone transplantation is regarded as the gold standard treatment strategy to restore bone defects after trauma, infections, tumour resection, or for nonunions (reviewed by Dimitriou, Mataliotakis, Angoules, Kanakaris, & Giannoudis, 2011). Nevertheless, the use of autologous bone grafts is associated with infections, size mismatch, and limited availability of donor tissue. Most importantly, an adverse effect of autologous bone grafting is the introduction of new skeletal defects, accompanied by donor site morbidity and chronic donor site pain in 28% of cases (Ross, Tacconi, & Miles, 2000). Future therapies for critical size bone defects would be a great improvement if they could eliminate the need for donor tissue while ideally maintaining the efficacy of autologous bone grafting. Regenerative medicine holds the promise to develop constructs outside of the body that can be created using the desired cell types, implanted at the defect site, and designed to meet the biomechanical demands.

The maximum size of vital engineered bone constructs has traditionally been limited by the poor diffusion of oxygen and nutrients to the core regions (reviewed by Carmeliet & Jain, 2000 and Rouwkema, Rivron, & van Blitterswijk, 2008). One potential strategy to solve this problem is to establish an integrated functional vascular network together with stimulation of osteogenesis and bone matrix formation, even without perfusion (reviewed by Liu, Chan, & Teoh, 2015 and Unger et al., 2007).

One approach to create a vascular network in engineered bone tissue is to coimplant cell populations that can establish vasculature and can differentiate into osteogenic cells. Prior studies have emphasized the mutual importance of multipotent mesenchymal stromal cells (MSCs) in supporting vasculogenesis and the significance of endothelial progenitor cells (EPCs) in the stimulation of the osteogenic potential of MSCs, both in vitro and in vivo (reviewed by Frohlich et al., 2008, Guerrero et al., 2013, Li & Wang, 2013, Liu et al., 2012, Unger et al., 2007). A specific type of EPC named “endothelial colony forming cells” (ECFCs), especially those derived from cord blood, showed robust proliferative potential and inherent vasculogenic and angiogenic capacity and can contribute to de novo blood vessel formation in vitro and in vivo, in contrast to mature ECs (reviewed by Banno & Yoder, 2018). However, only few studies have addressed the simultaneous formation of in vitro prevascular networks and osteogenesis with these type of cells (Gawlitta et al., 2012, reviewed by Liu et al., 2015, Liu et al., 2012).

Several key steps towards clinical translation remain to be taken. For example, the reproducibility and standardization of coculture protocols and outcomes of selected release criteria are essential in enabling quality control of the resulting prevascularized tissue constructs for clinical application. At present, it is not known how the choice and combination of cells from one or different donors affects coculture outcomes. Data regarding ECFC heterogeneity have only been rarely reported, whereas MSCs have been widely described to have heterogeneous characteristics, potentially influenced by isolation and culture methods, donor age, donor gender, and medical history (Bertram, Mayer, & Schliephake, 2005; Katsara et al., 2011; Phinney et al., 1999; Portalska et al., 2013; Schellenberg et al., 2011; Siddappa, Licht, van Blitterswijk, & de Boer, 2007; Stenderup, Justesen, Clausen, & Kassem, 2003). Nevertheless, the influence of heterogeneity of donor MSCs on their specific osteogenic potential has only been examined in monolayer culture models. Therefore, the first aim of the present study is to examine the effects of donor variation on the vasculogenesis and osteogenesis of a 3D coculture model of subcultured primary MSCs and ECFCs.

Translating 3D cocultures to the clinic by incorporation of (preferably) autologous cells raises another critical challenge: limited cell sources. Ideally, both MSCs and ECFCs would be derived from an autologous cell source to avoid an immunogenic response upon implantation. However, the isolation of ECFCs from the adult peripheral blood is inefficient compared with isolation from cord blood, which is usually an allogeneic cell source. To date, the use of (autologous) adult peripheral blood‐derived ECFCs is considered unfavourable in the clinical setting because the prevalence of ECFC is extremely low (20 times lower than in cord blood), resulting in very low isolation yields that hamper the reproducibility and viability of possible therapies (reviewed by Banno & Yoder, 2018, Mund, Estes, Yoder, Ingram, & Case, 2012). Moreover, the angiogenic potential of peripheral blood‐derived ECFCs appears substantially lower than that of their cord blood‐derived counterparts (Ingram, Mead, Tanaka, et al., 2004, reviewed by Richardson & Yoder, 2011). Alternatively, induced pluripotent stem cells (iPSCs) could provide an unlimited source of clinically relevant, autologous endothelial cells with vasculogenic capacity. Consequently, our second goal was to evaluate the use of iPSC‐derived endothelial cell precursors (iECs), differentiated from several independent iPSC lines in the Matrigel coculture model with MSCs, and assess their vasculogenic capacity and reproducibility in an osteogenic coculture model.

In the present study, a Matrigel coculture system in an osteogenic environment was developed in which donor dependency of vasculogenic and osteogenic cells and their behaviour could be assessed.

## 2 MATERIALS AND METHODS

### 2.1 Isolation of MSCs

MSCs were isolated from human bone marrow aspirates that were obtained from consenting patients (*n* = 7; aspiration procedure was approved by the local medical research ethics committee, University Medical Center Utrecht) that underwent different surgical procedures (indicated in Table 1). These isolates are referred to as MSC1 to MSC7. Researchers were blinded to the medical history of the donors.

**Table 1:** Overview of the multipotent mesenchymal stromal cell (MSC) donor specifics used in experimental set‐ups

**Donor**	**Age**	**Gender**	**Type of surgery**	**Indicated figure**
MSC1	32	Female	Spinal fusion	Figure 3, 4, 5
MSC2	64	Male	Total hip prosthesis	Figure 3 and Figure S4
MSC3	69	Male	Total hip prosthesis	Figure 3 and Figure S4
MSC4	41	Female	Total hip prosthesis	Figure 4 and 5
MSC5	60	Female	Spondolysis	Figure 4 and 5
MSC6	19	Female	Total hip prosthesis	Figure 2
MSC7	64	Male	Total hip prosthesis	Figure 2

Aspirates were diluted 1:1 with phosphate‐buffered saline (PBS) and filtered through a 100‐μm cell strainer. The mononuclear cell (MNC) layer was retrieved after centrifugation (415 × *g)* on a Ficoll‐Paque (GE Healthcare) gradient (density 1.077 g/ml). Approximately 250,000 MNCs were plated per cm^2^ in MSC expansion medium consisting of α‐Minimum Essential Medium (Gibco Paisley, 22561), supplemented with 10% heat‐inactivated fetal bovine serum (FBS; Hyclone CSG0412 or RYG35912), 100 U/ml penicillin and 100 μg/ml streptomycin (PenStrep, Gibco), 0.2 mM L‐ascorbic acid‐2‐phosphate (ASAP, Vitamin C; Sigma), and 1 ng/ml basic fibroblast growth factor (rh‐FGF‐2; R&D Systems). After their first passage, MSCs were either further expanded or frozen; cells from passage 4 were used in the experimental set‐ups. In experiments with iECs, constructs were made with commercially available human MSCs obtained from Lonza (Poietics Human MSC) as detailed in Section 2.5.

### 2.2 Isolation of ECFCs

Cord blood of seven different donors was used (procedure was approved by the medical research ethics committee, University Medical Center Utrecht, informed consent was obtained from the mothers) as a source for the ECFC isolation (hereafter referred to as ECFC1 to ECFC7; researchers were blinded to the condition of the mother and donor child). Cord blood was diluted with PBS (1:3), and the mononuclear cell layer was retrieved after centrifugation (400 × *g)* on 1.077 g/ml Ficoll‐Paque gradient (GE Healthcare). 20 × 10^6^ MNCs were plated in a 50‐μg/ml collagen type I‐coated (BD Biosciences, rat tail) well of a six‐well plate with 1 ml of complete endothelial growth medium‐2 (EGM‐2) containing Endothelial Basal Medium‐2 + SingleQuots (Lonza), 100 U/ml‐100 μg/ml PenStrep, and 10% heat‐inactivated FBS. The medium was changed daily until day 7 and then three times per week. Between weeks 2 and 4, ECFC colony outgrowth was observed. When individual colonies expanded, but did not touch each other yet, the cells were trypsinized and replated into collagen type I‐coated culture flasks at a density of ~7,000 cells/cm^2^. Complete EGM‐2 medium was used for subsequent cell expansion. After isolation, ECFCs were either expanded or frozen and used between passages 7 and 12.

### 2.3 Characterization of cell types

#### 2.3.1 Multipotent mesenchymal stromal cells (MSCs)

Multipotency of MSCs was examined via differentiation towards adipogenic, osteogenic, and chondrogenic lineages as described previously (Gawlitta et al., 2012). Briefly, osteogenesis was examined by staining for ALP activity (Vector SK5100 kit, Vector Laboratories) after culturing for 14 days under osteogenic differentiation medium (ODM), which consisted of α‐MEM (Gibco Paisley, 22561), 10% heat‐inactivated FBS, 0.2 mM ASAP, 100 U/ml‐100 μg/ml PenStrep,10 mM β‐glycerophosphate (Sigma), and 10 nM dexamethasone (Sigma).

Differentiation towards the adipogenic lineage was examined by staining for lipid droplets with Oil‐Red‐O in iso‐propanol after 21 days of culturing in adipogenic differentiation medium (ADM). ADM consisted of α‐MEM (Gibco Paisley, 22561), 10% heat‐inactivated FBS, 100 U/ml‐100 μg/ml PenStrep,1 μM dexamethasone, 0.5 mM 3‐isobutyl‐1‐methylxanthine (I7378, Sigma), 0.2 mM indomethacin (I5879, Sigma), and 1.72 μM insulin (I0516, Sigma).

Chondrogenic differentiation of the MSCs was examined by culturing them in aggregates of 250,000 cells per pellet for 3 weeks. The pellets were cultured in chondrogenic differentiation medium consisting of high glucose DMEM (Gibco Paisley, 31966), 1% Insulin‐Transferrin‐Selenium (ITS) + premix (BD Biosciences), 0.1 μM dexamethasone, 0.2 mM ASAP, 100 U/ml‐100 μg/ml PenStrep, and 10 ng/ml transforming growth factor β2 (TGF‐β2; R&D Systems). Medium was changed for the first 4 days daily, afterwards every 3 or 4 days.

MSCs were phenotypically characterized by cell surface marker expression profiles with flow cytometry (BD Canto II analzyer). Expression of CD90 (THY1, FITC‐conjugated; Abcam, ab124527), CD73 (AD2, PE‐conjugated; Abcam, ab157335), and CD105 (MEM‐226, APC‐conjugated; Abcam, ab60902) was confirmed, as well as the absence of CD34 (4H11, APC‐conjugated; Abcam, ab155377), CD45 (MEM‐28, PEC‐conjugated; Abcam, ab134202), CD97a (HM47, PE‐conjugated; Abcam, ab177274), and CD14 (RPA‐M1, FITC‐conjugated, Abcam, (ab86896). IgG‐matched controls were purchased from Abcam (APC, ab91358; PE, ab37392 and FITC, ab37393). Results show expression of the markers on cells based on FSC and SSC characteristics. Characterization of donor MSC6 is shown as a representative example (Figure S1).

#### 2.3.2 Endothelial colony forming cells (ECFCs)

Phenotypic characterization of ECFCs was performed using a BD FACSCanto II Flow Cytometer (BD Biosciences, Breda, the Netherlands). Cells were detached using accutase and checked for the following endothelial makers: anti‐hVEGFR2‐PE (R&D Minneapolis, MN), anti‐hVE‐Cadherin‐PE (R&D), anti‐CD31‐PE (R&D), anti‐CD90‐PE (R&D), anti‐CD105‐PE (R&D), anti‐CD34‐FITC (BD), anti‐CD90 AF647 (Biolegend), and anti‐CD133‐PE (Miltenyi, Bergisch Gladbach, Germany), as well as absence of haematopoietic/myeloid marker expression with anti‐CD45‐PE (BD) and anti‐CD14‐PE (Biolegend, San Diego, CA).

Additional characterization was performed by immunofluorescent staining. Cells were grown until confluency in chamber slides (Thermo Fisher, Landsmeer, the Netherlands), fixed with 4% formaldehyde and permeabilized with 0.1% Triton X‐100 where appropriate. Anti‐CD144 (R&D), anti‐CD31 (R&D), and anti‐von Willebrand Factor (vWF, Sigma) primary antibodies were used, secondary staining was performed with anti‐Mouse AF555 and anti‐rabbit AF488 secondary antibodies, and nuclei were visualized with 4′,6‐diamidino‐2‐phenylindole (DAPI). Images were taken with a Zeiss LSM700 Confocal Microscope. Fluorescent‐activated cell sorting (FACS) profiling was performed for one ECFC donor (Figure S2).

### 2.4 In vitro MSC‐ECFC cocultures in Matrigel

Cocultures were performed in growth factor‐reduced Matrigel (354230, BD Bioscience). The samples were prepared by mixing 50 μl ODM, containing both cell types, with 50 μl Matrigel. Each sample of 100 μl gel/ODM contained a total cell volume of 625,000 cells (ratio of 4:1 MSCs to ECFCs) and was pipetted into a 12‐well plate. The mixture was allowed to form a hydrogel at 37°C for at least 1 hr, resulting in a hemispherically shaped construct, after which 1 ml ODM was added on top. The medium was changed every 3–4 days. On day 10 or day 21, hydrogels were fixed with formalin (10%) and stored in PBS (4°C) until further processing.

To assess prevascular structure formation in large constructs, cocultures (MSC6/MSC7‐ECFC7) were molded in custom‐made cylindrical silicone molds (SYLGARD 184 Silicone Elastomer Kit, Dow Corning) measuring 5 mm diameter × 5 mm height. The cocultures were maintained in the molds with ODM on top. The constructs were removed from the molds after 4 days of culture. These constructs were then maintained in ODM until fixation on day 10.

Because of limitations in the numbers of competent subcultured primary cells, the influence of donor variation on vasculogenic and osteogenic potential had to be studied with different sets of MSC‐ECFC combinations. The combinations used to evaluate the influence on vasculogenesis and early osteogenesis (ALP) are displayed in Table 2 (three donors for each cell type, *n* = 3 gels per combination). Table 3 indicates the combinations used to study the influence of MSC and ECFC variation on the osteogenic properties of the constructs (i.e. osteonectin expression and mineralization) (*N* = 3 independent repetitions, *n* = 2 gels per combination).

**Table 2:** MSCs (M) and ECFCs (E), each derived from three different donors (Table 1) were cocultured in various combinations for assessment of vasculogenic differentiation

M1E1	M1E2	M1E3
M2E1	M2E2	M2E3
M3E1	M3E2	M3E3

*Note*. MSC: multipotent mesenchymal stromal cell; ECFC: endothelial colony forming cell.

**Table 3:** Overview of MSC (M) and ECFC (E) donor combinations for assessment of osteogenic differentiation

M1E4	M1E5	M1E6
M4E4	M4E5	M4E6
M5E4		

*Note*. MSC: multipotent mesenchymal stromal cell; ECFC: endothelial colony forming cell.

### 2.5 Generation of iECs from iPSCs and iEC/MSC cocultures

Endothelial cells were differentiated from human iPSCs and are referred to as iECs, as previously described (Barruet et al., 2016; White et al., 2013). Briefly, two iPSC lines (Control episomal iPS cell lines eWT‐1323‐2 and eWT‐BJ2; Matsumoto et al., 2013) were used and both differentiated twice into iECs; hereafter referred to as iEC1–1; iEC1–2 and iEC2–1; iEC2–2, respectively. FACS of KDR (VEGFR2) and PECAM (CD31) double positive cells (PECAM1‐AF488; clone M89D3 #558068 and KDR‐APC; clone 89106 #560871) was performed on the single cells derived from the embryoid bodies, as described in Theodoris et al. (2015). The sorted PECAM^+^/KDR^+^ cells were subsequently cultured on fibronectin‐coated plates with complete ECM (Sciencell). iECs were combined in coculture with commercially available MSCs obtained from Lonza (Poietics Human MSC; 24655, PT‐2501), and used between passages 6 and 10 in the cocultures. Cocultures were created and cultured as described in Section 2.4.

### 2.6 (Immuno)histochemical staining

#### 2.6.1 CD31, NG2, and α‐SMA staining in hydrogels

To demonstrate the presence of endothelial cells in the cocultures, CD31 immunofluorescence staining was conducted on half a sample fixed on day 10. After permeabilization, nonspecific protein binding was blocked before 1 hr of incubation with the primary anti‐CD31 antibody (0.13 mg/ml mouse anti‐human CD31, M0823, Dako), with or without anti‐NG2 chondroitin sulfate proteoglycan antibody (0.001 mg/ml, Merck Millipore, AB5320). After washing with 0.1% Tween in PBS, 1 hr of incubation with the secondary antibody (1:200, sheep anti‐mouse biotinylated, RPN1001v1, GE Healthcare) was performed. The samples were incubated with streptavidin Alexa Fluor 488, 2 mg/ml (Invitrogen), together with the anti‐α‐smooth muscle actin (α‐SMA) fluorescently labelled antibody (Clone 1A4, Cy3 0.5 μg/ml, C6198, Sigma Aldrich). The 5 mm Ø × 5 mm constructs were incubated overnight (4°C) with the antibodies. Finally, nuclei were counterstained with 4′,*6*‐*diamidino*‐*2*‐*phenylindole dihydrochloride* (DAPI) staining 100 ng/ml (Sigma) for 15 min at room temperature.

#### 2.6.2 CD31 staining on paraffin sections

Sections (5 μm) were cut from the samples (5 mm diameter × 5 mm constructs, the groups from Table 3 and iEC cocultures) after embedding in paraffin. Following dewaxing and rehydration steps, antigen retrieval was performed on the sections by boiling in citrate buffer (10 mM, pH 6) for 15 min, washing in demi water, and 0.1% Tween in PBS. Peroxidase blocking was carried out in 0.3% H₂O₂ in PBS (10 min). After blocking (15 min, 5% BSA/PBS), slides were incubated overnight at 4°C with either the anti‐CD31 primary antibody (0.13 mg/ml mouse anti‐human, Dako, M0823) or concentration‐matched isotype control (isotype mouse igG1, Dako, X0931). The samples were incubated with the secondary antibody (6.5 mg/ml rabbit anti‐mouse HRP, Dako, P0260) for 1 hr. Then they were exposed to the tertiary polymeric HRP‐linker antibody (brightVision poly‐HRP‐anti Rabbit, Immunologic DPVO55HRP) for 1 hr, followed by addition 3,3′‐diaminobenzidine (DAB) substrate solution which is transformed to brown staining facilitated by HRP. Optionally, the staining was combined with an α‐SMA staining (1:300 Monoclonal Anti‐Actin, α‐Smooth Muscle—Alkaline Phosphatase antibody, Sigma, clone 1A4), colour development with ALP‐kit (Vector SK5100 kit, Vector Laboratories) performed before boiling in citrate buffer. Nuclei were counterstained with Mayer's hematoxylin (Merck). Finally, the sections were dehydrated and mounted with Depex or aquamount.

#### 2.6.3 Osteocalcin/osteonectin staining on paraffin sections

Upscaled cylindrical constructs and iEC‐containing cultures were tested for osteogenic differentiation by staining for the osteoblast marker osteocalcin (OCN). Effects of variation of the MSC donor on osteogenic differentiation were examined via the osteogenic marker osteonectin (ON) in the set of cocultures in Table 3. ON production was determined after 10 and 21 days of culture. Peroxidase blocking was performed before the sections were boiled in citrate buffer (10 mM, pH 6) for 20 min. After protein blocking (15 min, 5% BSA/PBS), slides were incubated with the primary anti‐ON antibody (~9 ug/ml, DSHB, AON‐1 was deposited to the DSHB by Termine, J.D. (DSHB Hybridoma Product AON‐1)) and incubated at RT for 2.5 hr. Sections were washed three times for 5 min with PBS‐Tween, after which they were incubated with an anti‐mouse HRP‐labelled polymer for 1 hr (Dako, Envision, K400011). Addition of DAB substrate solution resulted in a brown staining, facilitated by HRP. Nuclei were counterstained with Mayer's hematoxylin (Merck). Finally, the sections were dehydrated and mounted with Depex.

The osteocalcin staining was performed according to a similar protocol with the following changes: Antigen retrieval was performed on the sections by incubation in pronase (Sigma, 10165921001; 30 min, 1 mg/ml) and hyaluronidase (Sigma, H2126; 30 min, 10 mg/ml). The incubation with primary antibody (0.02 mg/ml) mouse antibovine osteocalcin (clone OCG4, Takara M044) was performed overnight at 4°C. The sections were then incubated with the secondary antibody GAM‐HRP (5.5 mg/L goat anti‐mouse, Dako, P0447) for 1 hr.

#### 2.6.4 Von Kossa staining on paraffin sections

Von Kossa staining was used to visualize mineral deposition in the cocultures (Table 3) after 21 days of in vitro culture. Nondemineralized and rehydrated sections were incubated with 1% silver nitrate in demi water (Fisher Scientific) directly under a regular light bulb for 1 hr, prior to washing with 5% sodium thiosulfate in demi water (Alta Aesar). Mayer's hematoxylin nuclear counterstaining was performed before dehydration and mounting in Depex.

### 2.7 Alkaline phosphatase activity in hydrogels

ALP was used to indicate early osteogenesis, as the activity is associated with committed osteoprogenitor cells. For detection of ALP activity (in the samples shown in Table 2) on day 10, the fluorescent Vector SK5100 kit was used. In order to link the ALP activity with the vasculogenesis in one half of a construct, the remaining half of the construct was used for the CD31 staining (Table 2). Constructs were permeabilized and incubated with the kit's Red Substrate in the dark followed by washing in demi water.

### 2.8 Quantification of the prevascular structures

The influence of donor variation on angiogenic properties was further assessed by quantifying the total length of all CD31‐positive structures in the images (length in pixels). Images of the CD31‐stained cocultures (*n* = 3 per group) of Table 2 were captured (Olympus BX60, Cell‐F software) and processed in Adobe Photoshop CS6. The levels were individually adapted by eliminating over‐exposed and under‐exposed pixels following conversion of the images to black and white. Subsequently, the images were inverted (brightness −150 and contrast 100). The resulting files were batch processed in the freeware programme “Angioquant” (Niemisto, Dunmire, Yli‐Harja, Wei Zhang, & Shmulevich, 2005). All images had individually optimized processing settings for smoothening, segmentation with automatic thresholding, and pruning of structures below 25 pixels. Then, the total length of the prevascular structures (total length in one image of a construct at day 10) was assessed.

### 2.9 Statistical analyses

Statistical analyses were performed with GraphPad Prism 6.01. Angioquant outcomes were tested for the significant influence of donor variation on the total structure length with a two‐way ANOVA with multiple comparisons of the mean, when varying the donor of the MSCs or the ECFCs. A Tukey HSD Post hoc test was used to correct for multiple comparisons. Differences between the experimental groups were considered statistically significant if *p* < 0.05. Outcomes are presented as means ± SD.

## 3 RESULTS

### 3.1 Identification and characterization of MSCs and ECFCs

As expected, the bone marrow‐derived MSCs showed a fibroblast‐like morphology, their multi‐lineage potential was confirmed, and their CD‐marker expression profile was consistent with accepted criteria for MSCs (Dominici et al., 2006; Figure S1a–d). ECFCs organized into their characteristic colonies with rounded cell morphology and exhibited a cobblestone morphology upon passaging. The ECFCs showed high expression of endothelial/haematopoietic stem cells markers (Figure S2).

### 3.2 Formation of 3D prevascular structures in MSC‐ECFC osteogenic cocultures

**Figure 1:**
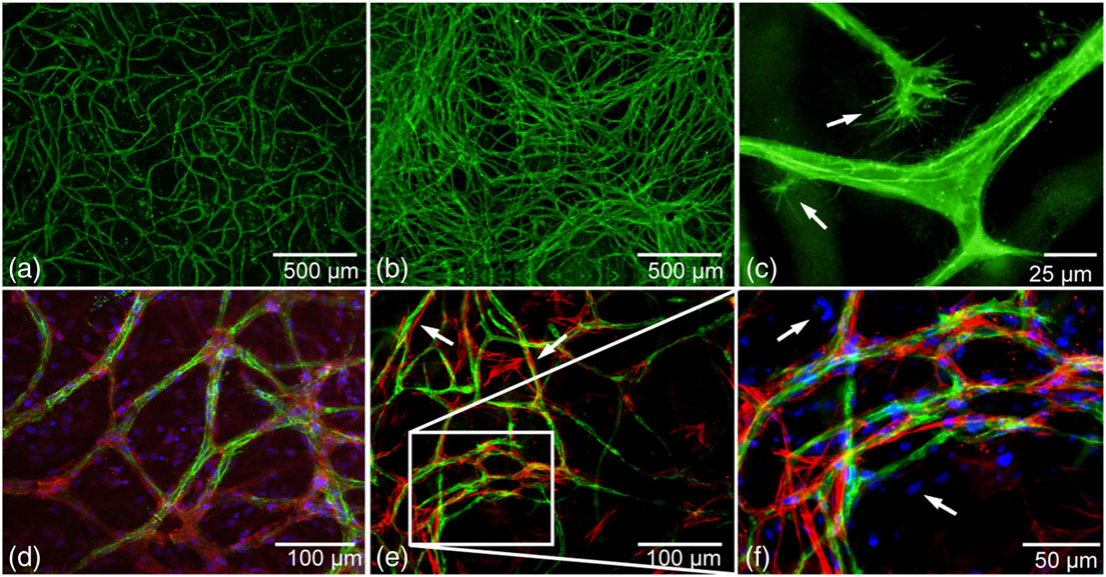
Prevascular structures and mural‐like cells in osteogenic MSC‐ECFC cocultures in Matrigel. CD31‐positive staining in green shows endothelial structures after 10 (a) and 21 (b) days in Matrigel cultures in osteogenic differentiation medium. (c) Sprouting tip cells were observed with filopodia (arrows), (d) NG2 positive cells (red) align with the prevascular structures (CD31, green), and (e) α‐SMA‐positive cells (red, arrows), supported the endothelial structures (green, CD31). (f) Higher‐magnification image from (e) showing cell nuclei (blue, arrows) of cells that did not stain for CD31 (green) or α‐SMA (red). Images are representative of at least three independent experiments with various MSC and ECFC donor combinations [Colour figure can be viewed at wileyonlinelibrary.com]

Hemispherical 100 μl‐sized cocultures of MSCs with ECFCs in Matrigel, cultured in osteogenic differentiation medium, produced CD31‐positive 3D pre‐vascular networks by day 10 (Figure 1a). At day 21, an even more extensive network with an increased number of junctions was observed (Figure 1b). Pseudopodial processes, on sprouting tip cells, were observed in the constructs at both day 10 and 21, indicating ongoing angiogenesis (Figure 1c). In addition, pericytic mural cells adjoining the CD31‐positive prevascular structures were visualized by NG2 (Figure 1d) and α‐SMA detection (Figure 1e).

The nuclear staining of the cocultures demonstrated that not all cells contributed to vasculogenesis or differentiated towards mural cells (Figure 1f). Most likely, these cell nuclei belong to cells which are committed to the osteogenic lineage (as presented in Figure S4). Monocultures of either MSCs or ECFCs did not show prevascular network formation or α‐SMA‐positive network formation (Figure S3).

### 3.3 Cocultures in larger 3D constructs

**Figure 2:**
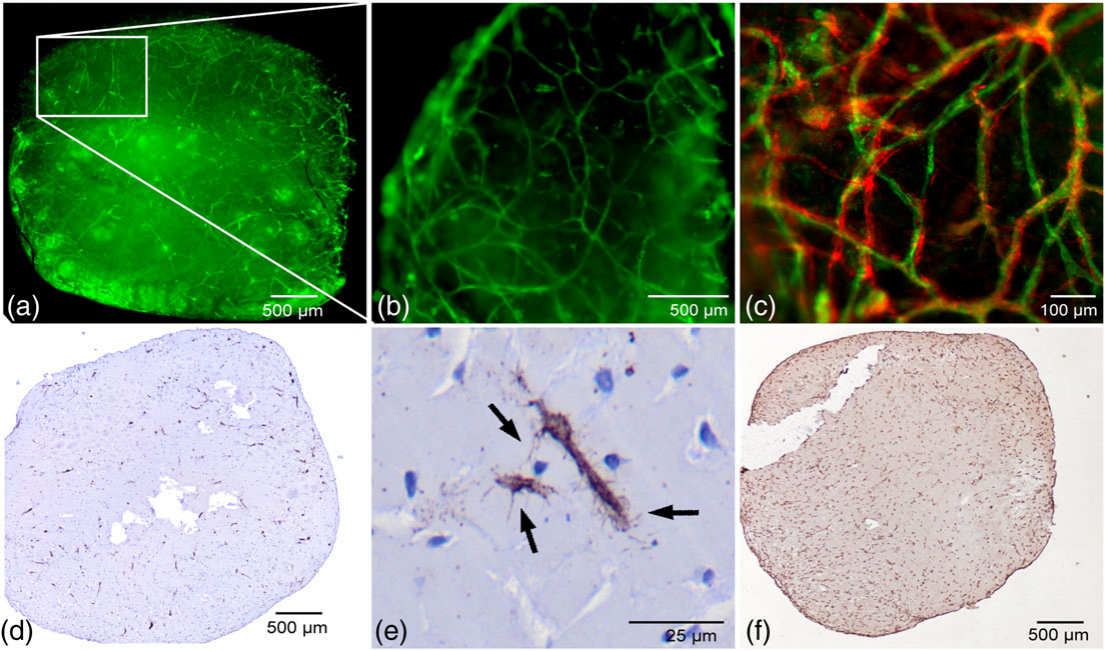
Vasculogenesis and osteogenesis in larger MSC‐ECFC 3D Matrigel constructs at day 10. (a,b) CD31‐positive structures were found throughout the volume of the 5 mm Ø × 5 mm Matrigel whole‐mount constructs, (c) adjoined by α‐SMA‐positive cells. (d,e) On sections, CD31 staining shows prevascular network formation and sprouting cells, and (f) osteocalcin deposition (brown) was observed throughout the complete cross‐section of the construct. Images are representative of two independent experiments with two MSC donors with one ECFC donor (M6E7 and M7E7) [Colour figure can be viewed at wileyonlinelibrary.com]

To confirm 3D vascular network formation in constructs of a considerable size, 5 mm Ø × 5 mm cylindrical‐shaped MSC‐ECFC osteogenic Matrigel cocultures were established. Prevascular structures were recognized throughout these well‐shaped cylindrical constructs (Figure 2a–e). Cross‐sectional images from the centre of the construct showed similar structures as observed in the 100 μl‐sized, thin samples of Figure 1, including the α‐SMA‐lined endothelial structures (Figure 2c). In addition, osteogenic differentiation of cells in these large constructs was demonstrated by positive OCN staining throughout the construct (Figure 2f).

### 3.4 The effects of donor variation on prevascular network formation

**Figure 3:**
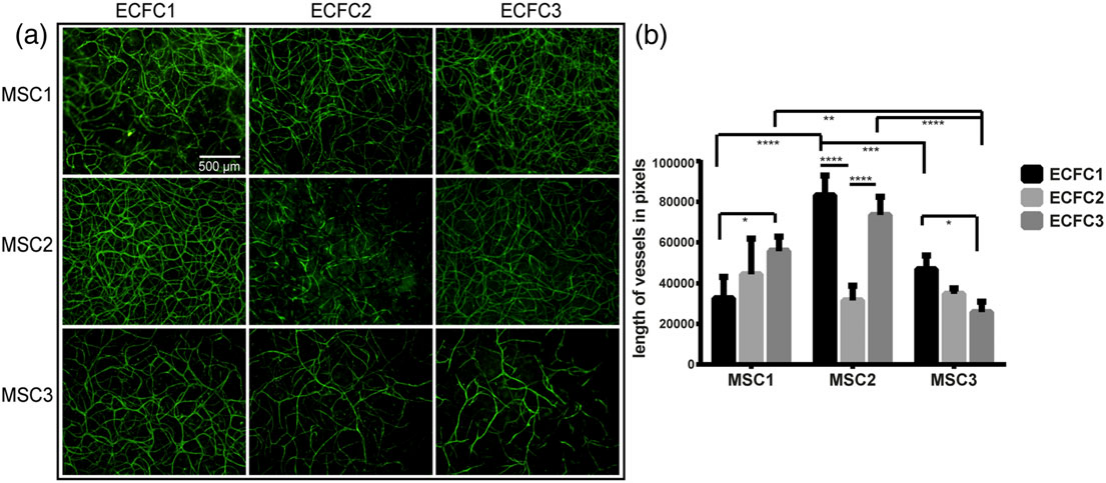
Influence of multipotent mesenchymal stromal cell (MSC) and endothelial colony forming cell (ECFC) donor variation on the formation of prevascular structures. (a) The prevascular networks formed by nine donor combinations (Table 2) are shown at day 10 (CD31 staining in green). The scale bar represents 500 μm for all images in (a). Images are each representative of triplicates. (b) Quantification of the prevascular structures revealed significant differences in total structure length (pixel number) among the donor combinations. ^*^ *p* < 0.05, ^**^ *p* < 0.01, ^***^ *p* < 0.001, ^****^ *p* < 0.0001 [Colour figure can be viewed at wileyonlinelibrary.com]

All evaluated donor combinations (Table 2) of MSCs with ECFCs, cultured in 100 μl‐sized hemispherical Matrigel constructs produced a CD31‐positive 3D prevascular network by day 10 (Figure 3a). Nonetheless, the total length of the vascular structures (in pixels) was significantly different in the various combinations, indicating that the angiogenic properties differed when varying the donor combinations (Figure 3b). More specifically, when varying the ECFC donor in the coculture combinations, the total vessel length was affected within one MSC donor group for some donor combinations (Figure 3b) (M1E1 *vs* M1E3; M2E1 *vs* M2E2; M2E2 *vs* M2E3; and M3E1 *vs* M3E3). Similarly, by varying MSC donor while the ECFC donor was unchanged, a significant difference in the prevascular network length was observed (*e.g.,* M1E1 *vs* M2E1 and M2E1 *vs* M3E1). From this, it can be concluded that both the MSCs' and ECFCs' donor individually influence the length of the prevascular structures, thus the angiogenic properties of the coculture. Overall, the specific donor combination of MSC‐ECFC rather than one cell type has the overhand in determining the length of the pre‐vascular network; that is, the cell types react differently when combined with cells from different donors.

### 3.5 Osteogenic differentiation in MSC‐ECFC cocultures

To confirm simultaneous osteogenic differentiation and vasculogenesis in the cocultures, the constructs analyzed in Figure 3 were also evaluated for ALP activity. Qualitative analysis confirmed that the nine coculture combinations displayed ALP activity throughout the construct (Figure S4). Among these combinations, minimal variation in the intensity of the ALP staining was visible. An increase of ALP activity was found in MSC‐ECFC cocultures compared with the corresponding MSC monocultures (Figure S4, insets).

**Figure 4:**
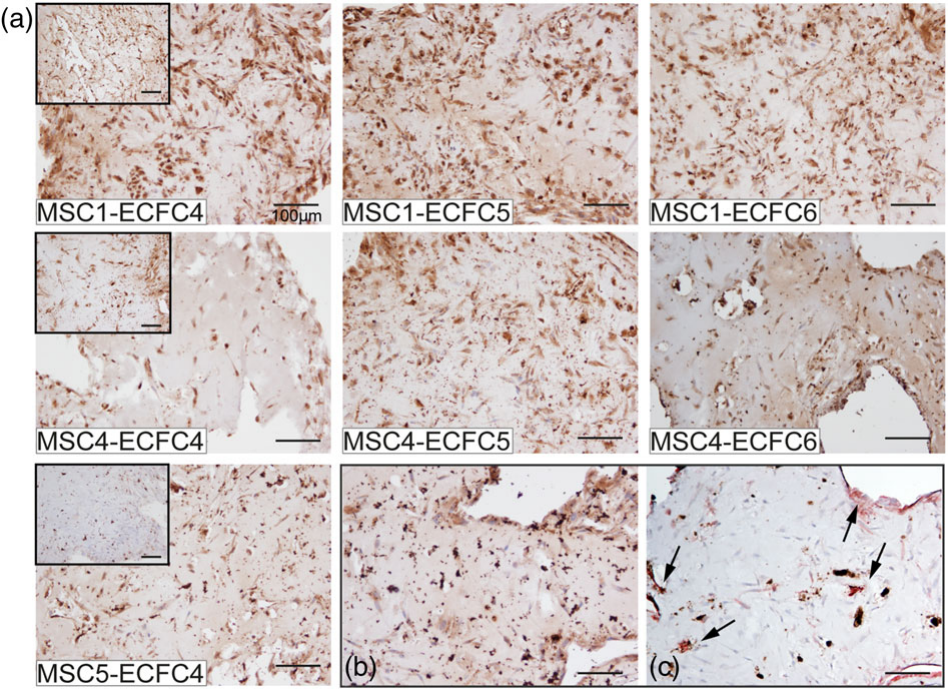
Osteonectin deposition in MSC‐ECFC cocultures in Matrigel at day 21. (a) Cocultures from three different MSC donors and from three ECFC donors revealed that variation of the ECFC donor did not strongly affect ON deposition at day 21, as well as the variation of the MSC donor. Insets contain the corresponding ON‐stained MSC monoculture controls, where a difference in ON staining intensity can be appreciated. Images are representative of three independent experiments (*n* = 2/experiment). (b) ON protein expression is shown in brown, with the corresponding (c) CD31^+^ vascular structures (brown) and αSMA positive mural cells (red) as detected in consecutive slides of donor combination M5E4. Black arrows indicate positive staining. Scale bar represents 100 μm [Colour figure can be viewed at wileyonlinelibrary.com]

To further confirm osteogenic differentiation in the cocultures, ON expression was evaluated in the MSC‐ECFC combinations described in Table 3. Figure 4 shows the ON expression in the cocultures after 21 days, as well as for MSC monoculture controls (Figure 4, insets). In all combinations, ON expression was detected after 10 days (Figure S5), with an increase in cellular and matrix staining intensity after 21 days of culture. Matrigel constructs containing MSC monocultures showed variable ON expression (Figure 4a, insets). Addition of ECFCs to the MSC‐containing constructs did not have an explicit influence on this ON deposition at day 21. In constrast, at day 10, the cocultures showed more ON expression than to the corresponding MSC monocultures. Also, cocultures stained for ON at 10 days showed formation of cell clusters, positive for ON, which may be indicative of early bone nodule formation and corresponding osteoblast maturation (Qu, Rausch‐Fan, Wieland, Matejka, & Schedle, 2007).

No clear influence of variation of the MSCs donor or ECFCs donor was found on the ON expression at both time‐points 10 or 21 days. A relatively small difference in ON expression could be observed among MSC monocultures. After addition of ECFCs (independent of the donor), an increase in ON staining instensity on protein level was observed. Simultaneous osteogenic differentiation and vasculogenesis in one construct was confirmed by expression of ON and CD31/αSMA on consecutive slides of donor combination M5E4 (Figure 4b,c).

**Figure 5:**
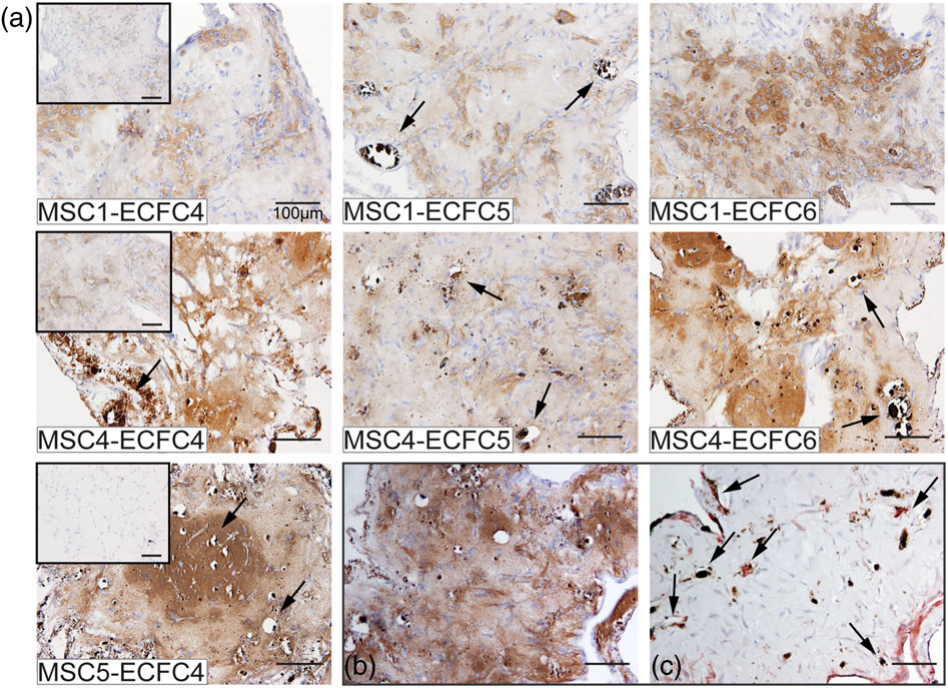
Mineralization in MSC‐ECFC cocultures in Matrigel at day 21. (a) The images show increased von Kossa staining (brown) in the cocultures, compared with corresponding MSC monocultures (insets) in all cultures. Variation of the ECFC donor did not influence the extent of mineralization. Variation in the MSC donor did influence the extent of mineralization (M1E4, M4E4, M5E4 and M1E6, and M4E6). Mineralization in brown is shown with the corresponding (c) CD31^+^ vascular structures (brown) and αSMA positive mural cells (red) as detected in consecutive slides of donor combination M5E4. Black arrows indicate positive staining. Images are representative of three independent experiments (*n* = 2/experiment). Scale bar in images represents 100 μm [Colour figure can be viewed at wileyonlinelibrary.com]

To determine if the osteogenic differentiation also resulted in construct mineralization, von Kossa staining was performed on day 21 on the donor combinations of Table 3 (Figure 5). All cocultures showed staining indicating mineralization, but only in selected combinations calcified nodule formation could be observed already. MSC1 only showed mineralized nodule formation when combined with ECFC donor 5, but was solely developed in a late stage during culture (observed via bright‐field microscopy, data not shown). In contrast, MSC4 contained calcified nodules when combined with all ECFC donors. Nodule formation for MSC4‐containing combinations was already observed at day 10 of culture, however formation of new nodules did not continue over the period of culture for M4E5. MSC5 containing cocultures also showed early nodule formation that increased over time, resulting in a highly mineralized matrix and nodule formation (Figure 5a). The combinations with mineralized nodules and matrix (M4E4—M4E6–M5E4) also showed a homogeneous staining of the matrix in the ON staining (Figure 4a). Interestingly, in corresponding MSC monocultures, the MSCs did not display mineralization by day 21 (Figure 5, insets). Overall, the extent of mineralization was determined mostly by the choice of MSC donor. In contrast, the variation of the ECFC donor did only influence the level of mineralization in the MSC1 coculture constructs; in all cases, ECFC addition did induce (a faster) mineralization. Presence of CD31/αSMA‐double positive structures in a mineralized matrix was confirmed by positive staining on consecutive slides of donor combination M5E4 (Figure 5b,c), on the same location as presented in Figure 4b,c.

### 3.6 Implementation of iECs/MSCs in Matrigel cocultures

**Figure 6:**
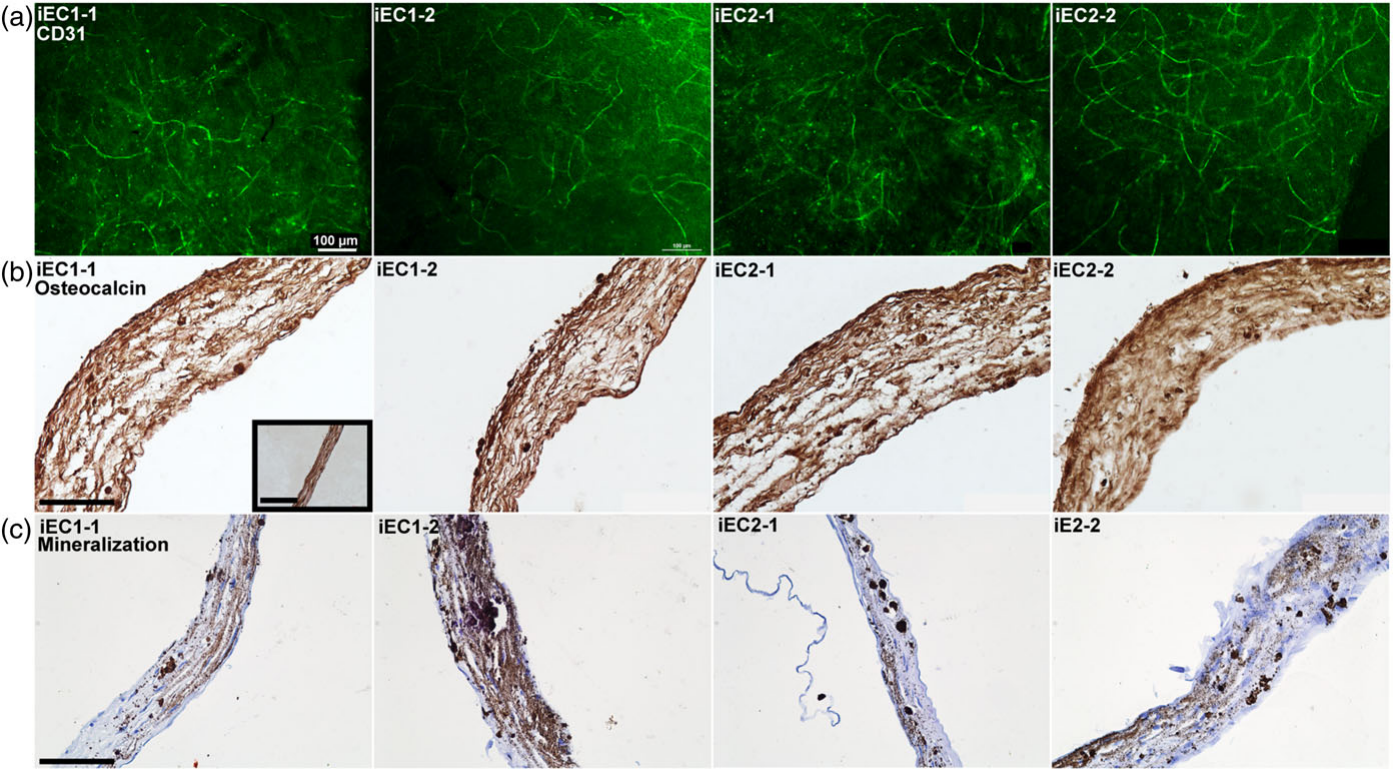
Effects of induced pluripotent stem cell‐derived endothelial (iEC) variation in iEC‐MSC cocultures on vasculogenesis and osteogenic differentiation. Cocultures of MSCs with iECs support (a) CD31‐positive endothelial network formation (green); (b) deposition of osteocalcin (brown; inset is MSC monoculture); and (c) mineralization (brown) in all combinations with highest mineral content in iEC1‐2. All cocultures used Poietics Human MSCs. Images are representative of four independent experiments. Scale bars in images represent 100 μm; scale bar in insets represents 50 μm [Colour figure can be viewed at wileyonlinelibrary.com]

Finally, we evaluated if human iECs could be used in 3D cocultures, if they would show de novo vasculogenesis, and if different iECs showed variable reproducibility in angiogenesis and/or osteogenic support in cocultures. CD31 staining after 10 days of coculture revealed that the four combinations of iECs with MSCs (two separate derivations of iECs from two iPSC lines) showed comparable levels of vasculogenesis when reviewed qualitatively (Figure 6a). Also, α‐SMA‐positive cells (Figure S6a) and CD31‐positive cells (Figure S6b) could be detected throughout the constructs via immunohistochemistry. OCN staining of the same constructs demonstrated that the four combinations of iEC‐MSC were all capable of undergoing osteogenesis (Figure 6b). Additionally, nodule mineralization of the constructs was confirmed (Figure 6c). The cocultures of the different combinations mineralized where the group with iEC1–2 showed the most intense staining. Overall, all cocultures containing iECs could successfully undergo vasculogenesis and support osteogenesis, suggesting that different iEC lines can show reproducible outcomes.

## 4 DISCUSSION

Current in vitro tissue engineering strategies are largely confined to the creation of small constructs. Generation of an inherent functional 3D vascular network within the tissue‐engineered construct is considered essential for the creation of larger constructs. Here, we used a matrigel/hydrogel‐based model to create vascular networks in an osteogenic construct, and used these constructs to investigate an important aspect of robustness of experimental outcomes.The model consistently generated 3D prevascular networks supported by mural cells in osteogenically differentiated constructs. This phenomenon was also observed when larger constructs were generated, in which the size reached the often described diffusion limit of 200 μm (reviewed by Carmeliet & Jain, 2000, and Rouwkema et al., 2008). The addition of ECFCs to the MSCs enhanced the osteogenic differentiation and mineralization. In contrast to most coculture models (reviewed by Liu et al., 2015), no endothelial cell stimulants were added to the culture medium, which suggests that induction of vasculogenesis can occur via cross‐communication of the embedded MSCs. We found that under the influence of osteogenic factors a subpopulation of the MSCs was committed to the osteogenic lineage, whereas other subpopulations of the MSCs and/or ECFCs supported endothelial network formation by differentiation into mural cells.

This finding is in line with other reports of successful vasculogenesis in cocultures in osteogenic medium (Gawlitta et al., 2012, reviewed by Liu et al., 2015, Liu et al., 2012). Our results indicate an extensive context‐dependent communication between MSCs and endothelial cells, creating a mutually stimulating environment likely involving (growth) factors and/or cell–cell contacts, influencing both mural cell differentiation, and vasculo‐angiogenic and osteogenic processes (Bidarra et al., 2011, reviewed by Hirschi, Ingram, & Yoder, 2008, Loibl et al., 2014, Nassiri & Rahbarghazi, 2014).

A close interplay between these growth factors and the opposing receptors signifies the balance in communication between the discussed cell types. When deriving the cell types from different donors, this delicate balance in communication is likely to be affected by donor variability. Accordingly, variations in outcomes of the vascular length and level of osteogenic differentiation were observed in our cocultures (Figures 3, 4, and 5). Here, we showed that the specific MSC‐ECFC combination, rather than one cell type, had the overhand in determining the levels of vasculogenesis. Furthermore, the MSC donor was a decisive factor in the amount and rate of osteogenic differentiation/mineralization in the coculture model. As such, an (im)balance in the cross‐communication might be linked to the influence of donor variation on vasculogenesis and osteogenesis.

Another explanation for the variation in osteogenic differentiation levels is that MSCs of different donors can react differently to dexamethasone that is present in the used osteogenic medium, resulting in diverging ALP expression, different expression profiles of osteogenic markers and mineralization (Kyllonen et al., 2013; Ogston, Harrison, Cheung, Ashton, & Hampson, 2002; Siddappa et al., 2007). This also matches previous observations of Siddappa et al., (2007) and Phinney et al., (1999), where a number of parameters were identified, such as the passage number and time point of medium change, that could additionally influence the maturation of the tissue‐engineered (osteogenic) constructs. This variability in steroid responsiveness may also affect the MSCs' communication with the ECFCs. Thus, variable reactions of MSCs to osteogenic medium can alter the osteogenic differentiation rate, but could also influence their signalling to the ECFCs.

Important aspects of translation towards clinically relevant constructs that consist of multiple cell types, as presented here, are the balanced cross‐communication, reproducibility of their performance, but also the limitation of cell sources and amount of cells that can be isolated can cause setbacks. Cord blood‐derived ECFCs have shown to be superior to the ECFCs isolated from peripheral blood in their proliferative and neoangiogenic capacity and are present in sufficient amounts in cord blood. Despite these promises, there are limitations in its use as an autologous cell source for adults because it requires biobanking of the cell product directly after birth. On the other hand, because the isolation of adult ECFCs is hampered by their low prevalence in peripheral blood, they could hardly serve as a robust autologous cell source for therapies for all patients in a clinical setting (reviewed by Banno & Yoder, 2018). As an alternative, patient‐specific iPSC (or HLA superdonor)‐derived ECs can serve as an unlimited source of clinically relevant cells (Cellular Dynamics International Inc, 2015). The coculture model in this study allowed the creation of constructs containing iECs, which were shown to be a feasible alternative for ECFCs. Our data provide proof of concept of reproducible implementation of iECs in osteogenic differentiation models and showed its potential of de novo blood vessel formation in an in vitro coculture setting. Hints of robustness of this approach are demonstrated by the similarity in differentiation of four different iECs with MSCs of one donor. In the future, the effect of donor variation on differentiation in coculture models could potentially be diminished by incorporation of ECs and MSCs derived from a single iPSC line of the patient.

Thus, by deriving iECs from patient‐derived iPSCs, the drawbacks of using cord blood‐derived ECFCs can potentially be overcome. Moreover, iPSCs exhibit an unlimited proliferation potential and therefore present an attractive (autologous) cell source for future regenerative treatments (Samuel et al., 2013). Furthermore, iECs might be able to reduce variation as iPSC‐derived endothelial cells were shown to be produced with high batch uniformity (Belair et al., 2015) and less variance than primary ECs (White et al., 2013), which has also been demonstrated in our iEC‐MSC cocultures (Figure 6).

So far, several research groups have reported successful creation of prevascular structures derived from iPSCs. iECs have been shown to repopulate decellularized tissue‐engineered vascular structures (Margariti et al., 2012), (self)assemble into perfusable tubular structures (Belair et al., 2015; Zanotelli et al., 2016), and form 3D networks in vitro (Chan et al., 2015; Orlova et al., 2014) and in vivo *(*Samuel et al., 2013). Orlova et al., (2014) succeeded in coculturing iPSC‐derived ECs and iPSC‐derived pericytes in a 2D environment, and demonstrated that iPSC‐derived ECs were able to functionally integrate into embryonic zebrafish vasculature. Nevertheless, before iPSCs can be integrated in therapies in clinical settings, safety concerns must be addressed.

Overall, our study demonstrates that care should be taken when varying donors of cells in coculture models as donor variation can affect cell differentiation and thus the reproducibility of results. It is imperative that several important hurdles towards clinical translation of functional prevascularized bone constructs are taken. This does not only include the choice of (autologous/allogeneic) cell sources but also upscaling of size, reproducibility, and standardization of (co)culture protocols and release criteria. Our in vitro 3D coculture model is an accessible method to explore new regenerative strategies to overcome these hurdles and move towards in vivo applications.

## CONFLICT OF INTEREST

The authors have declared that there is no conflict of interest.

## Supporting information


**Figure S1.** *Characterization of the Multipotent Mesenchymal Stromal Cells from bone marrow (MSC6)*. The primary human MSCs were found to be positive for the stem cell markers CD90, CD73, and CD105, and negative for CD31, CD34 and CD45. The MSC was shown to be able to differentiate into all 3 lineages (osteogenic – ALP; Adipogenic – oil Red O; chondrogenic – Safranin O).

**Figure S2.** *Characterization of the Endothelial Colony Forming Cells after isolation from cord blood*. With flow cytometry, the ECFCs were found to be positive for endothelial/haematopoietic stem cell markers CD31, CD34, KDR, CD90, CD133, VE‐Cadherin, and CD105 and negative for CD14 and CD45 (blue, red histograms display isotype controls). Also, immunofluorescent staining confirmed the presence of VE‐cadherin, CD31 and vWF protein (bottom row, respectively), the top row shows isotype staining controls.

**Figure S3.** *α‐Smooth muscle actin and CD31 positive cells in mono‐ and co‐cultures in Matrigel*. Mono‐culture controls did not exhibit endothelial network organization. (A) At day 3, structures were observed by light microscopy in co‐cultured constructs, but also in mono‐cultures of MSCs in Matrigel at day 3. (B) Stainings for CD31 (green) and α‐SMA (red) revealed that co‐cultures contained endothelial networks with adjoined α‐SMA‐positive cells while the structures in MSC mono‐cultures were only α‐SMA positive at day 10. In addition, the Matrigel cultures of ECFCs alone did not show any structure formation.

**Figure S4.** *Alkaline Phosphatase (ALP) staining in nine MSC‐ECFC donor combinations (*Table 2 *) after 10 days of culture*. All donor combinations showed ALP activity (red) to a similar extent. Images are representative of the triplicates and ALP activity of corresponding MSC mono‐culture controls can be found in the insets.

**Figure S5.** *Osteonectin protein expression in co‐cultures at day 10*. MSCs show ON expression when cultured alone in 3D Matrigel cultures in ODM. Mono‐cultures show a slight difference between donors (insets). Addition of ECFCs enhanced the expression of ON slightly and resulted in clusters of cells (black arrows).

**Figure S6.** *α‐Smooth muscle actin and CD31 positive cells in iEC‐MSC Matrigel co‐cultures*. (A) Representative paraffin sections of the iEC1–2 co‐culture showed α‐SMA positive structures (in red). (B) iEC1–1 co‐cultures show CD31 positive staining in paraffin sections.
