# Supplementary figures and images for: Effect of donor variation on osteogenesis and vasculogenesis in hydrogel cocultures
Source: J Tissue Eng Regen Med. 2019 Feb 8;13(3):433–45. doi: 10.1002/term.2807 (PMC6593839; doi:10.1002/term.2807)

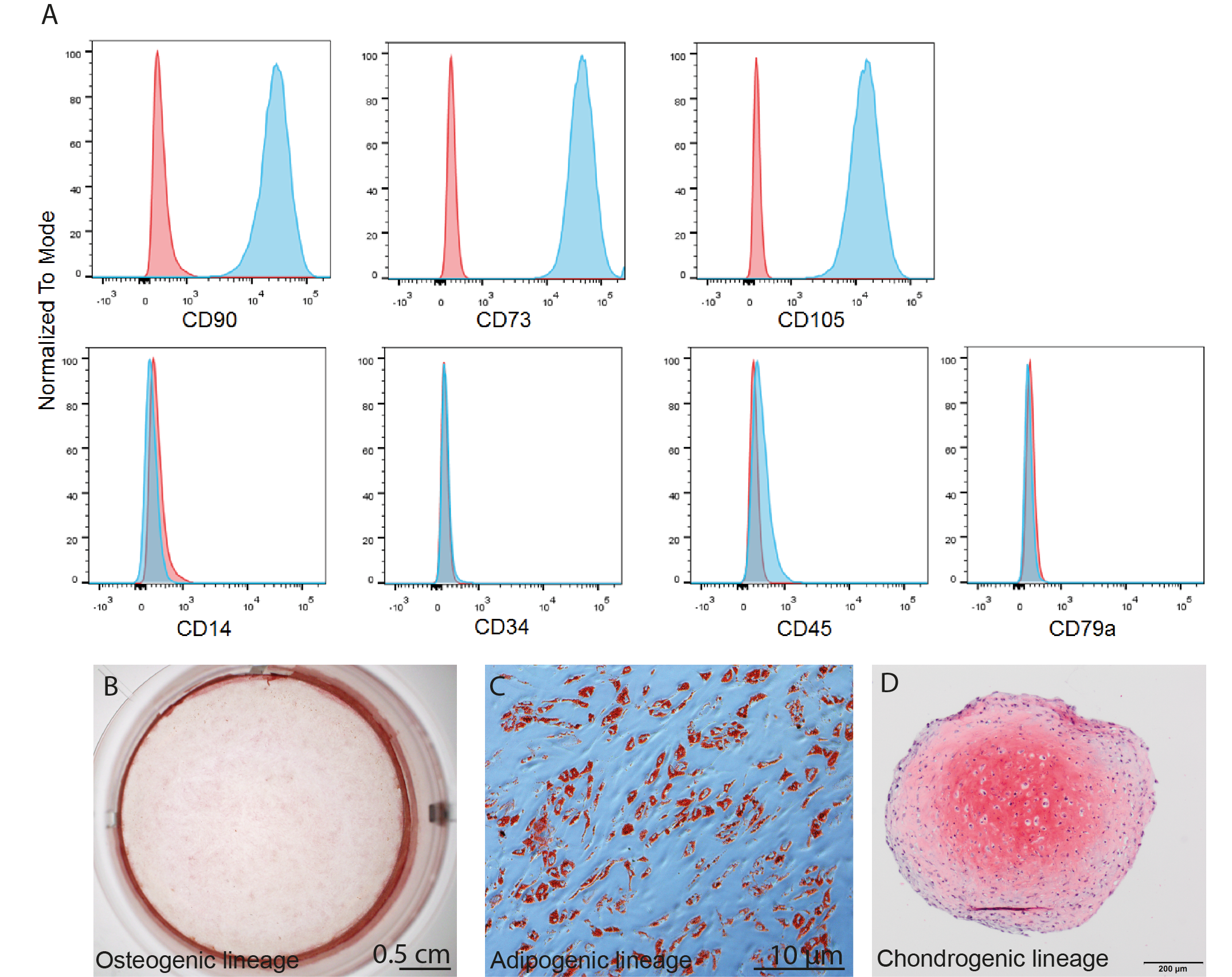

Supplement: Supplementary file 1 — Figure S1. Characterization of the Multipotent Mesenchymal Stromal Cells from bone marrow (MSC6). The primary human MSCs were found to be positive for the stem cell markers CD90, CD73, and CD105, and negative for CD31, CD34 and CD45. The MSC was shown to be able to differentiate into all 3 lineages (osteogenic – ALP; Adipogenic – oil Red O; chondrogenic – Safranin O). [file TERM-13-433-s001.tif]

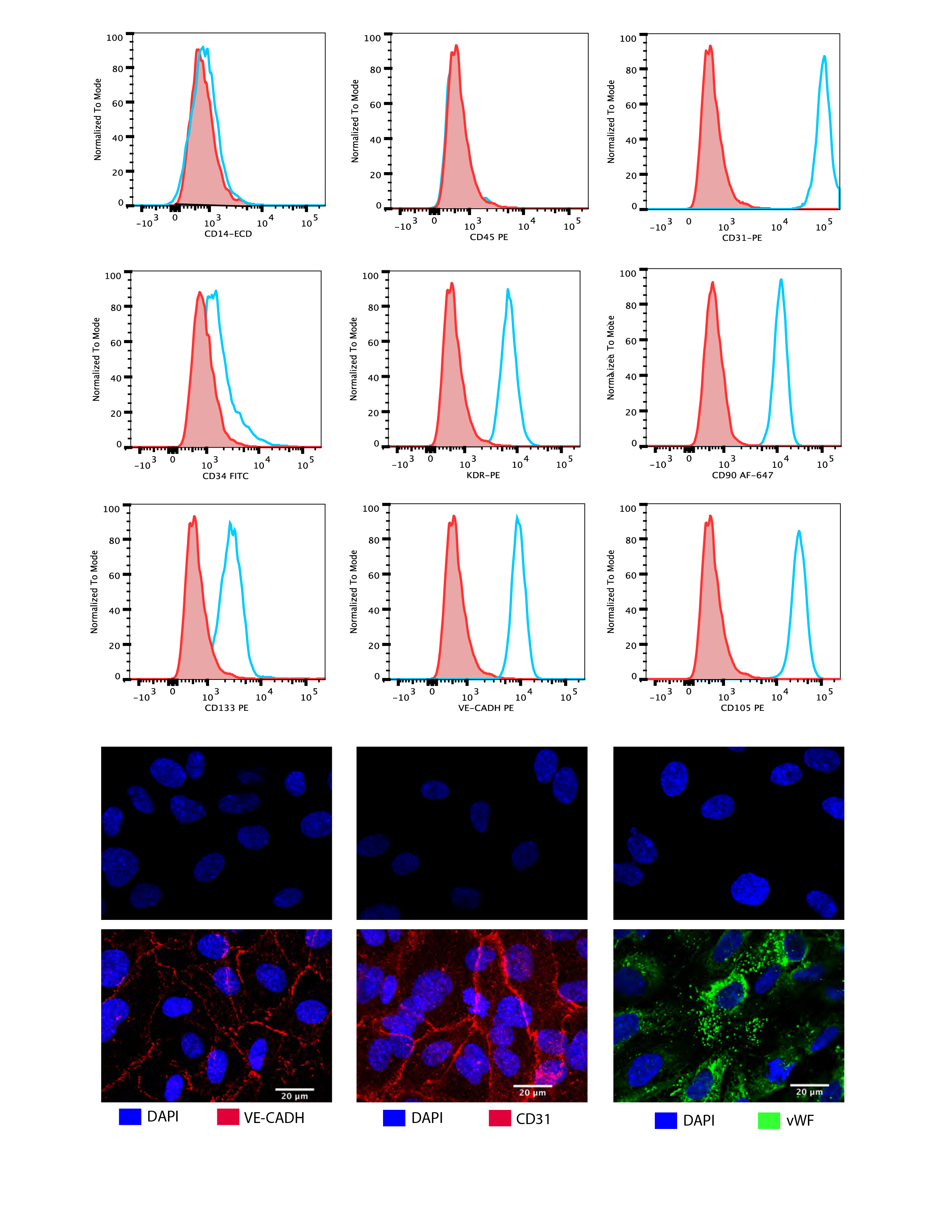

Supplement: Supplementary file 2 — Figure S2. Characterization of the Endothelial Colony Forming Cells after isolation from cord blood. With flow cytometry, the ECFCs were found to be positive for endothelial/haematopoietic stem cell markers CD31, CD34, KDR, CD90, CD133, VE‐Cadherin, and CD105 and negative for CD14 and CD45 (blue, red histograms display isotype controls). Also, immunofluorescent staining confirmed the presence of VE‐cadherin, CD31 and vWF protein (bottom row, respectively), the top row shows isotype staining controls. [file TERM-13-433-s002.tif]

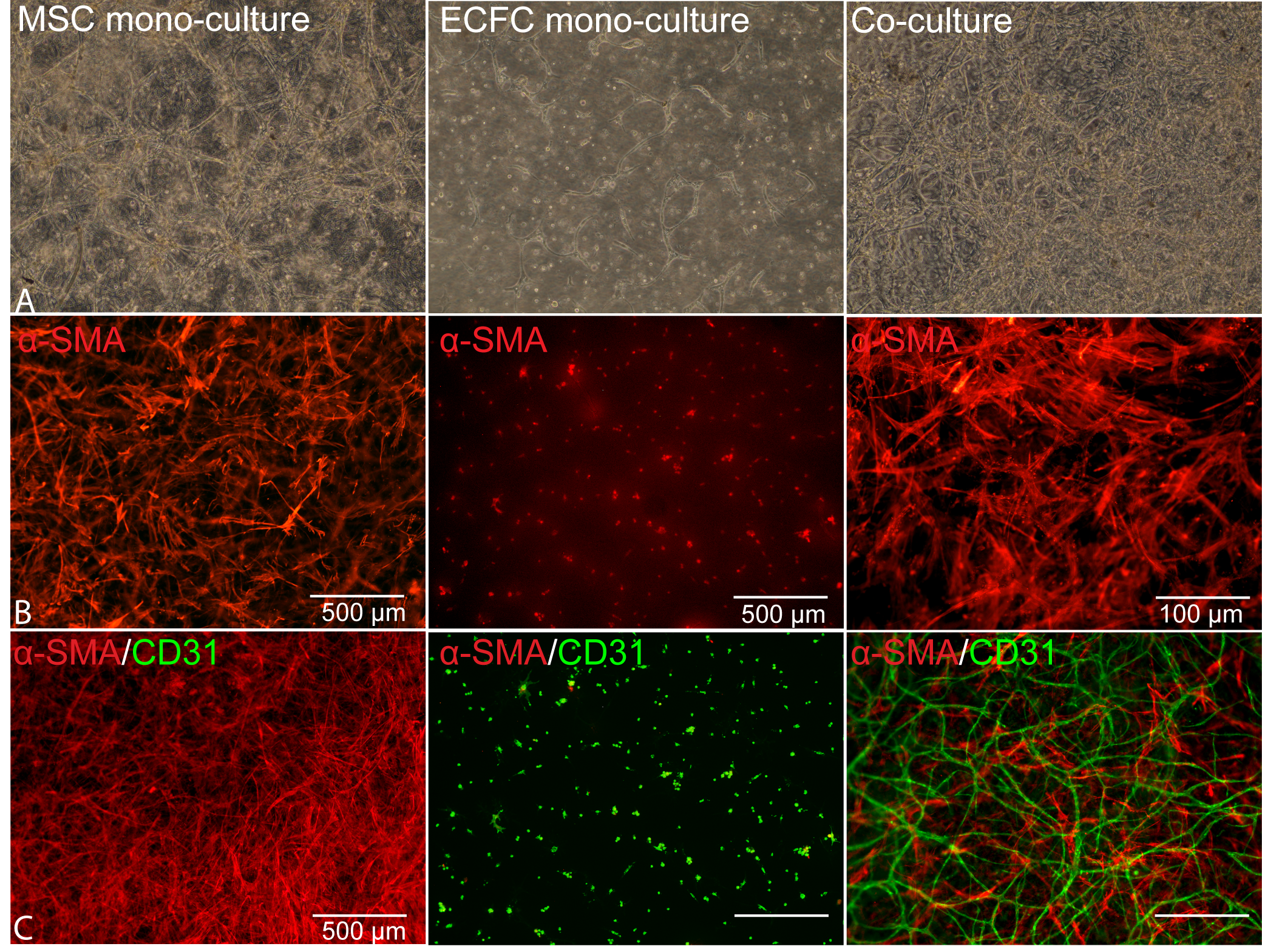

Supplement: Supplementary file 3 — Figure S3. α‐Smooth muscle actin and CD31 positive cells in mono‐ and co‐cultures in Matrigel. Mono‐culture controls did not exhibit endothelial network organization. (A) At day 3, structures were observed by light microscopy in co‐cultured constructs, but also in mono‐cultures of MSCs in Matrigel at day 3. (B) Stainings for CD31 (green) and α‐SMA (red) revealed that co‐cultures contained endothelial networks with adjoined α‐SMA‐positive cells while the structures in MSC mono‐cultures were only α‐SMA positive at day 10. In addition, the Matrigel cultures of ECFCs alone did not show any structure formation. [file TERM-13-433-s003.tif]

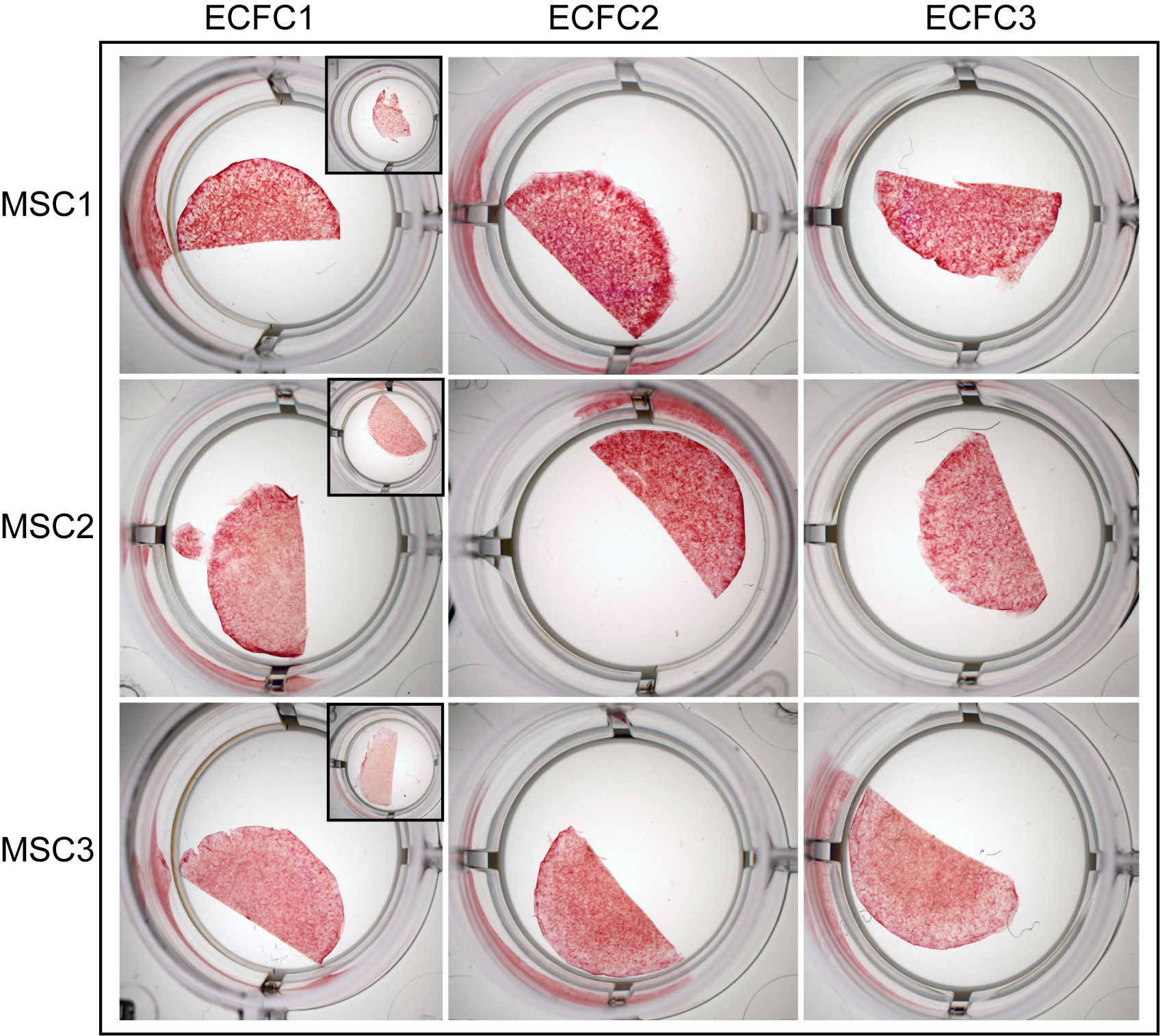

Supplement: Supplementary file 4 — Figure S4. Alkaline Phosphatase (ALP) staining in nine MSC‐ECFC donor combinations (Table 2 ) after 10 days of culture. All donor combinations showed ALP activity (red) to a similar extent. Images are representative of the triplicates and ALP activity of corresponding MSC mono‐culture controls can be found in the insets. [file TERM-13-433-s004.tif]

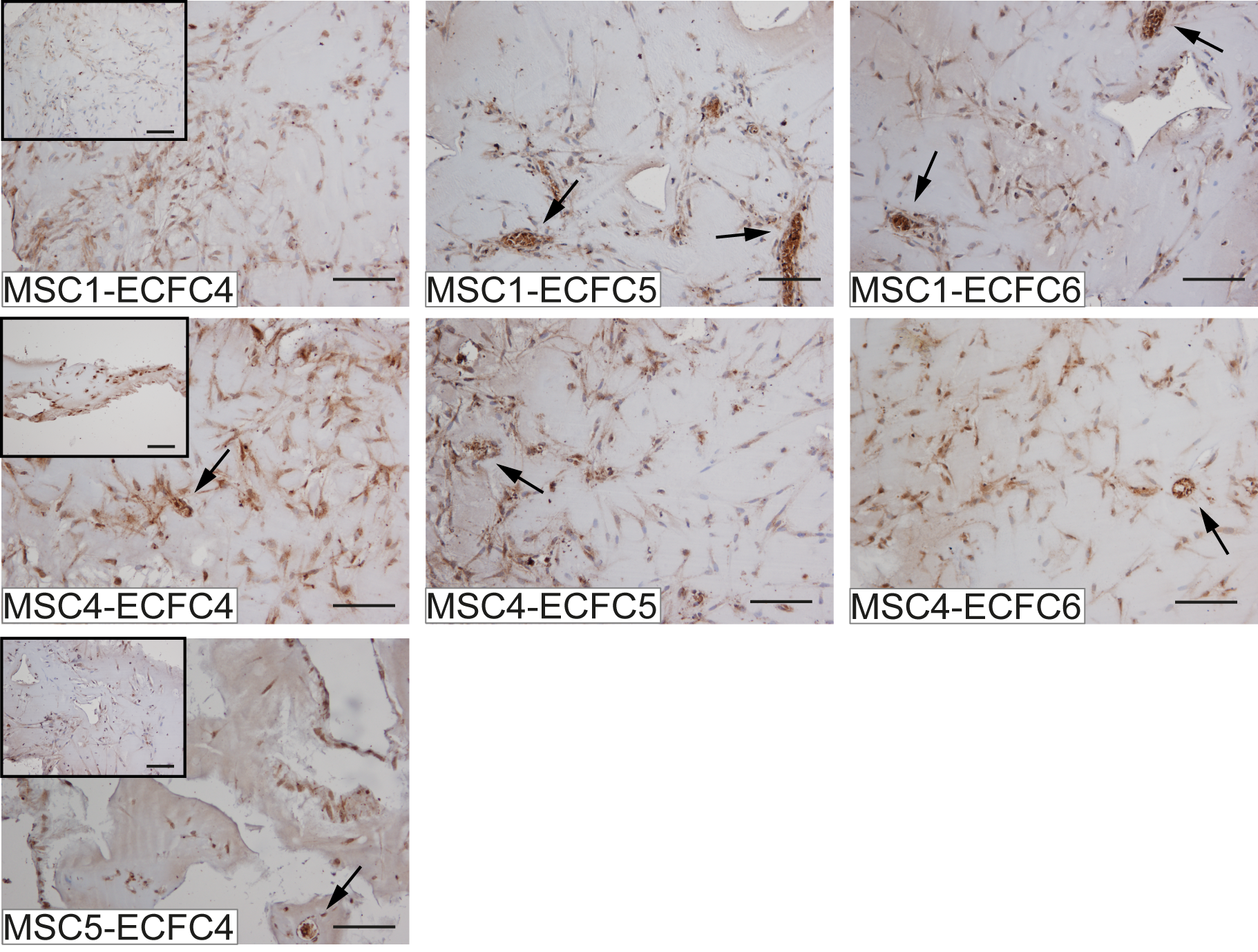

Supplement: Supplementary file 5 — Figure S5. Osteonectin protein expression in co‐cultures at day 10. MSCs show ON expression when cultured alone in 3D Matrigel cultures in ODM. Mono‐cultures show a slight difference between donors (insets). Addition of ECFCs enhanced the expression of ON slightly and resulted in clusters of cells (black arrows). [file TERM-13-433-s005.tif]

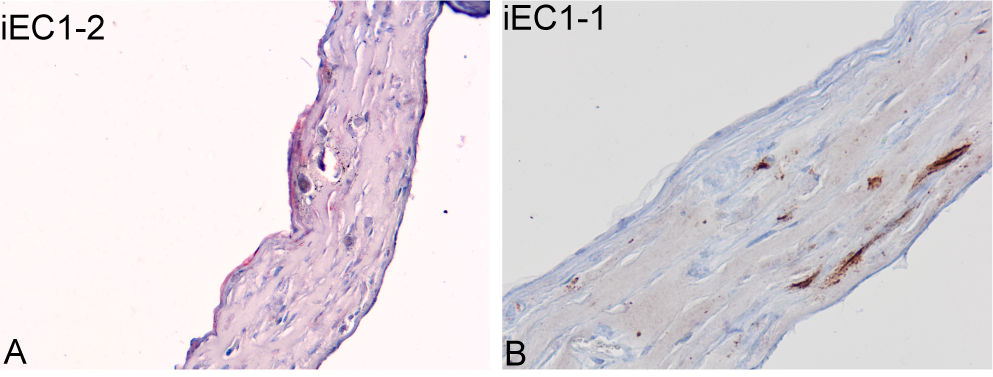

Supplement: Supplementary file 6 — Figure S6. α‐Smooth muscle actin and CD31 positive cells in iEC‐MSC Matrigel co‐cultures. (A) Representative paraffin sections of the iEC1–2 co‐culture showed α‐SMA positive structures (in red). (B) iEC1–1 co‐cultures show CD31 positive staining in paraffin sections. [file TERM-13-433-s006.tif]
